# Whole genome sequencing identifies missense mutation in *MTBP* in Shar-Pei affected with Autoinflammatory Disease (SPAID)

**DOI:** 10.1186/s12864-017-3737-z

**Published:** 2017-05-04

**Authors:** Julia Metzger, Anna Nolte, Ann-Kathrin Uhde, Marion Hewicker-Trautwein, Ottmar Distl

**Affiliations:** 1 Institute for Animal Breeding and Genetics, University of Veterinary Medicine Hannover, Foundation, Bünteweg 17p, 30559 Hannover, Germany; 2 Department of Pathology, University of Veterinary Medicine Hannover, Foundation, Bünteweg 17, 30559 Hannover, Germany

**Keywords:** Shar-Pei, Autoinflammatory disease, Whole genome sequencing, MTBP

## Abstract

**Background:**

Autoinflammatory diseases in dogs are characterized by complex disease processes with varying clinical signs. In Shar-Pei, signs of inflammation including fever and arthritis are known to be related with a breed-specific predisposition for Shar-Pei Autoinflammatory Disease (SPAID).

**Results:**

Clinical and histopathological examinations of two severely SPAID-affected Shar-Pei revealed signs of inflammation including fever, arthritis, and perivascular and diffuse dermatitis in both dogs. A multifocal accumulation of amyloid in different organs was found in one SPAID-affected case. Whole genome sequencing resulted in 37 variants, which were homozygous mutant private mutations in SPAID-affected Shar-Pei. Nine SNVs with predicted damaging effects and three INDELs were further investigated in 102 Shar-Pei affected with SPAID, 62 unaffected Shar-Pei and 162 controls from 11 different dog breeds. The results showed the missense variant *MTBP*:g.19383758G > A in *MTBP* to be highly associated with SPAID in Shar-Pei. In the region of this gene a large ROH (runs of homozygosity) region could be detected exclusively in the two investigated SPAID-affected Shar-Pei compared to control dog breeds. No further SPAID-associated variant with predicted high or moderate effects could be found in genes identified in ROH regions. This *MTBP* variant was predicted to affect the MDN2-binding protein domain and consequently promote proinflammatory reactions. In the investigated group of Shar-Pei older than six years all dogs with the mutant genotype A/A were SPAID-affected whereas SPAID-unaffected dogs harbored the homozygous wildtype (G/G). Shar-Pei with a heterozygous genotype (G/A) were shown to have a 2.13-fold higher risk for disease development, which gave evidence for an incomplete dominant mode of inheritance.

**Conclusions:**

The results of this study give strong evidence for a variant in *MTBP* related with proinflammatory processes via MTBP-MDM2 pathway. Thus, these results enable a reliable detection of SPAID in Shar-Pei dogs.

**Electronic supplementary material:**

The online version of this article (doi:10.1186/s12864-017-3737-z) contains supplementary material, which is available to authorized users.

## Background

High-throughput technologies have been shown to play an increasingly important role in clinical research and diagnostics [1–4]. In whole genome sequencing data various causative variants for disease traits have been detected in human as well as other mammals including cattle, horse and dogs [5–8]. It has been shown that disease causing mutations can be breed or population specific and are often linked or promoted by characteristic features or phenotypic traits supported in breeding programs [9, 10]. In Shar-Pei dogs, the development of a characteristically strongly wrinkled skin stabilized by deposits of hyaluronic acid were suggested to be involved in inflammatory processes resulting in a periodic fever [11]. It was proposed that a 16.1 Kb duplication upstream of the *hyaluronan synthase 2 (HAS2)* on chromosome (CFA) 13, which was detected in Shar-Pei with a padded muzzle (meatmouth type), was associated with skin wrinkling and periodic fever in Shar-Pei dogs [11, 12]. Therefore, this variant was assumed to affect both fever and thick and folded skin [11]. However, a direct relation of specific copy numbers detected by real-time PCR to Shar-Pei fever as an individual phenotype with no regard to other inflammatory signs could not be confirmed [13]. Further investigations of affected Shar-Pei showed that clinical manifestation was much more variable and recurrent bouts of fever were only one of various clinical signs designated now as a syndrome termed Shar-Pei autoinflammatory disease (SPAID) [14]. Additional signs of inflammation could be shown to be arthritis, dermatitis, otitis and systemic amyloidosis [14]. It was proposed that genetic risk factors on CFA13 and CFA14 might trigger these inflammatory processes [14]. Further droplet digital PCR (ddPCR) analysis of the 16.1 Kb copy number variation (CNV) upstream of *HAS2* revealed stable CNV alleles in this region in contrast to continuous alleles previously detected by real-time PCR and subsequently suggested an association with SPAID [15]. This breed specific CNV was suggested to be inherited in a bi-allelic way and proposed to explain 9.7% of the genetic variance whereas the influence of further potential causative variants and triggers for SPAID were still unclear [15]. The complexity of the disease and the variable onset of signs of inflammation showed a high similarity to human autoinflammatory diseases like the Familial Mediterranean Fever [16, 17]. Various genes have been shown to be involved in human autoinflammatory processes and were also found to interact with each other in triggering recurrent episodes of inflammation [18–23].

In our study, we investigated genetic variants derived from next generation sequencing (NGS) data to identify potential causative mutations which might be involved in the development of autoinflammatory processes and thus play a significant role in triggering SPAID in Shar-Pei.

## Results

### Phenotype

**Fig. 1:**
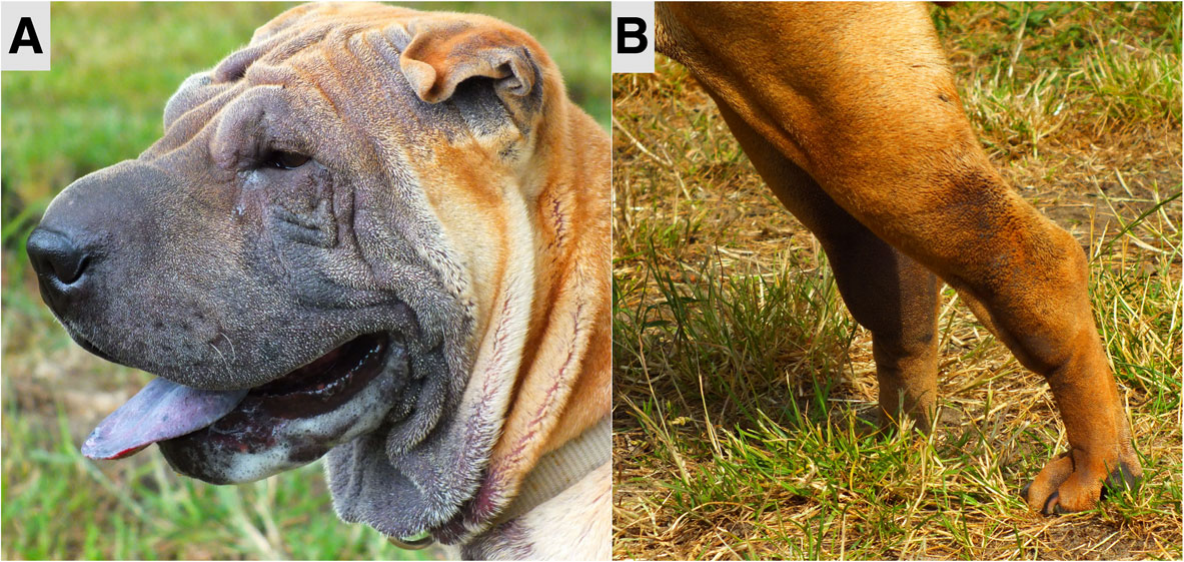
Exemplary illustration of clinical signs for SPAID. Affected Shar-Pei can show skin erythema in the region of wrinkles (**a**) or signs of inflammation at the tarsal joints (**b**)

Clinical investigations and patient histories recorded by a questionnaire resulted in 102 Shar-Pei with clinical signs consistent with SPAID and 62 unaffected dogs. The study included Shar-Pei of different breed types including horse, brush and bear coats, modern types with a meatmouth and traditional types with a bonemouth as well as Shar-Pei with a strongly wrinkled skin and others with few wrinkles (Table 1). In total, 46 of the SPAID-affected and 11 SPAID-unaffected Shar-Pei were older than six years and were therefore considered to be a reliable set for the SPAID-phenotype. First signs of the disease could be observed in a range of one to twelve years in the individual dogs whereas the average onset of the disease was 2.7 years (median 1.0). Only three Shar-Pei developed first signs of inflammation at an age of older than six years. Specific investigations of SPAID signs revealed all combinations of the inflammatory types fever, arthritis, erythema, thickened and pasty skin, otitis, eye inflammation and recurrent intestinal inflammations (Fig. 1, Additional file 1: Table S1).

**Table 1:** Phenotypes of 164 Shar-Pei investigated for SPAID

Age		≤6 years		>6 years	
Suspected SPAID- type		SPAID-affected	SPAID-unaffected	SPAID-affected	SPAID-unaffected
Breed specific phenotype					
Type	bonemouth	10 (17.86%)	10 (19.61%)	7 (15.23%)	5 (45.46%)
	meatmouth	46 (82.14%)	41 (80.39%)	39 (84.78%)	6 (54.54%)
Coat	horse	10 (17.86%)	9 (17.65%)	11 (23.91%)	5 (45.46%)
	brush	40 (71.49%)	31 (60.78%)	28 (60.87%)	4 (36.36%)
	bear	1 (0.56%)	1 (0.51%)	2 (4.35%)	0 (0.00)
	unknown	5 (8.93%)	10 (19.61%)	5 (10.87%)	2 (18.18%)
Wrinkles	few	11 (19.64%)	25 (49.02%)	17 (36.96%)	10 (90.91%)
	moderate	40 (71.49%)	25 (49.02%)	23 (50%)	1 (0.11%)
	pronounced	5 (8.93%)	1 (0.51%)	6 (13.04%)	0 (0.00)
Total number of individuals		56	51	46	11

Shar-Pei of different types concerning coat, mouth and degree of wrinkles in the age groups ≤6 years and >6 years used for this study are shown

### Histopathologic examination

**Fig. 2:**
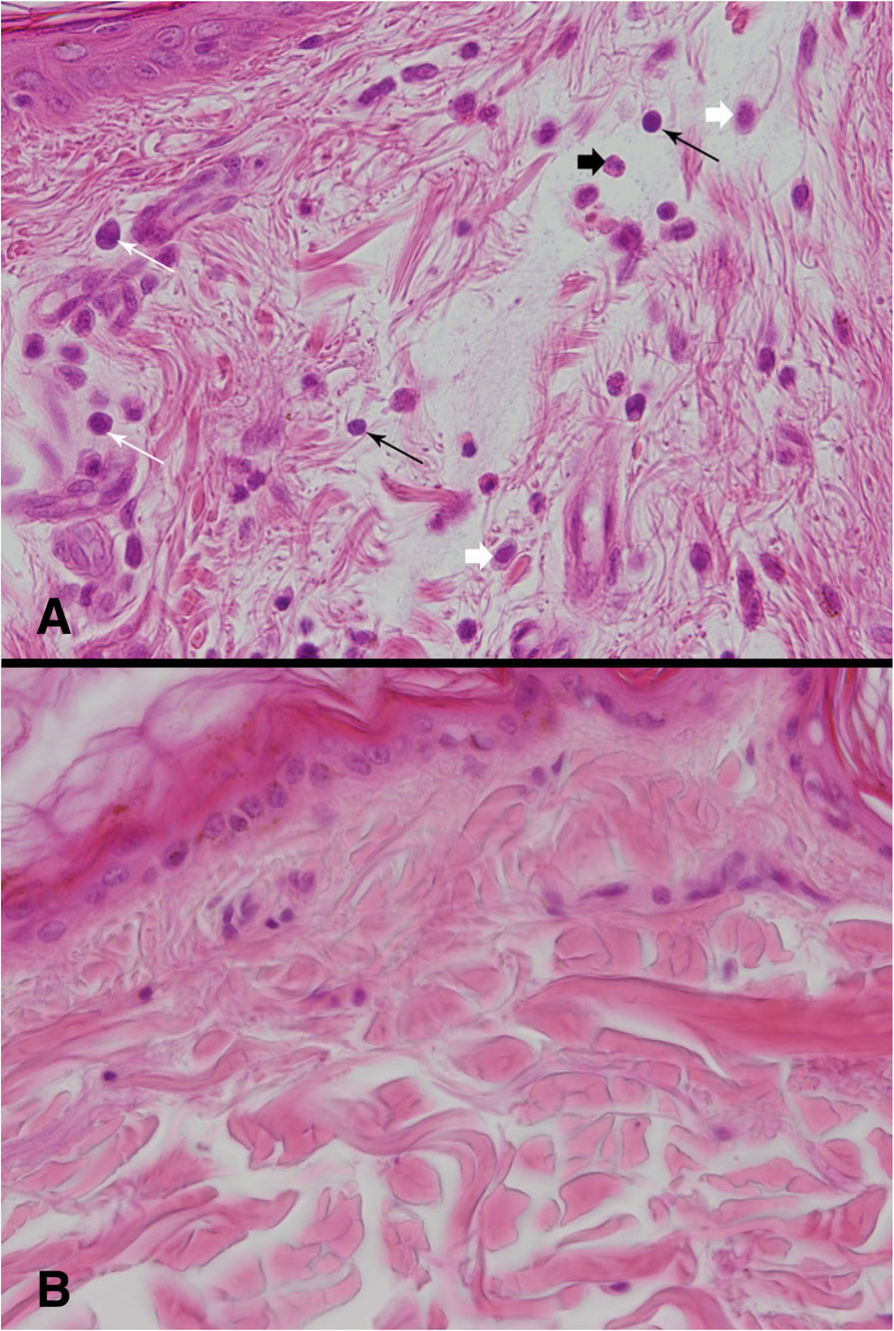
Inflammatory processes in the skin. Perivascular and diffuse infiltration with lymphocytes (long black arrows), plasma cells (long white arrows), eosinophilc granulocytes (short white arrow) and mast cells (short black arrows) in the skin of a SPAID-affected Shar-Pei (**a**). Normal skin of a SPAID-unaffected Shar-Pei (**b**). HE, magnification 400x

**Fig. 3:**
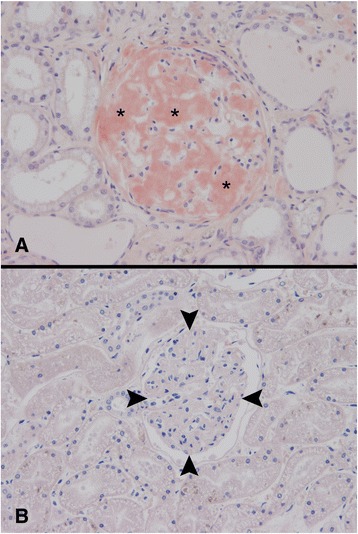
Histopathologic examination of the kidney. Accumulation of amyloid (asterisks) in a glomerulum of a SPAID-affected Shar-Pei (**a**). Normal glomerulum (arrow heads) of a SPAID-unaffected Shar-Pei (**b**). Congo red, magnification 200x

**Fig. 4:**
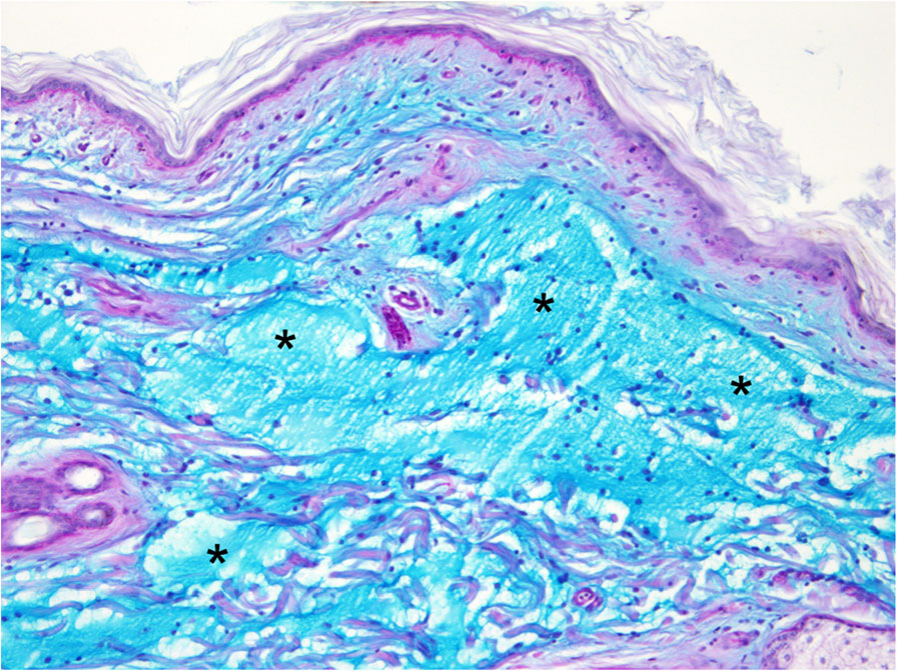
Cutaneous mucinosis. Marked dermal accumulations of alcian blue positive material (asterisks) in a SPAID-affected Shar-Pei. AB/PAS, magnification 100x

Due to bad general condition and poor prognosis two severely SPAID-affected Shar-Pei were euthanized and underwent histopathological examination. In addition, an SPAID-unaffected dog with no signs of inflammation was used as control. That dog was euthanized because of poor prognosis due to metastasizing tumors. In both SPAID-affected dogs, the skin revealed a perivascular and diffuse dermatitis with lymphocytes, plasma cells, eosinophilic granulocytes and mast cells (Fig. 2). In addition, one SPAID-case revealed a multifocal accumulation of amyloid in the salivary gland, liver, pancreas, kidneys (Fig. 3), adrenal glands, thyroid glands, spleen and in the lamina propria mucosae of stomach, small and large intestine. In all three examined Shar-Pei a generalized cutaneous mucinosis was present (Fig. 4).

### Whole genome sequencing

The whole genome of the two severely SPAID-affected Shar-Pei with recurrent bouts of fever and acute joint inflammations confirmed by careful examination of an accredited veterinarian was sequenced in order to compare genetic variants for an association with SPAID using data from five reference dogs of different breeds from sequence read achieve (SRA). Due to the high incidence of SPAID-affected progeny and parents, these two investigated Shar-Pei were expected to be very likely homozygous carriers of a potential causative mutation for SPAID. Whole genome sequencing resulted in a coverage of 11X (Shar-Pei 1) and 12X Shar-Pei 2 (Additional file 2: Table S2). In total, 31 missense variants, two disruptive in-frame insertions and deletions, one noncoding transcript exon variant, two frameshift variants and one splice acceptor variant could be detected in filtering analysis for homozygous mutant genotypes exclusively present in SPAID-affected Shar-Pei (Table 2). Six SNVs that were predicted to be deleterious (SIFT) as well as probably damaging (PolyPhen-2) and three insertions/deletions (INDELs) were chosen for further investigation of their association with SPAID (Table 3).

**Table 2:** Results from filtering analysis for SPAID-associated genotypes

CFA	Position	Base change	Amino acid change	Consequence	Genotype (5 reference dogs)	Genotype (2 affected Shar-Pei)	Gene (transcript)	SIFT	PolyPhen-2
1	8186638	A > G	K > R	missense variant	0/0	1/1	RTTN (ENSCAFT00000000055)	tolerated (0.3)	benign (0.036)
1	112413056	T > C	K > E/K > E	missense variant	0/0	1/1	CD79A (ENSCAFT00000037106/ENSCAFT00000008000)	tolerated low confidence	benign (0.227)
1	112413114	C > CGTGATG	I67_T68dup/I67_T68dup	disruptive inframe insertion	0/0	1/1	CD79A (ENSCAFT00000037106/ENSCAFT00000008000)	NA	NA
3	14027517	A > C	L > R	missense variant	0/0	1/1	ARSK (ENSCAFT00000012646)	deleterious low confidence	probably damaging (0.993)
4	6374801	G > A	C > Y	missense variant	0/0	1/1	PCNXL2 (ENSCAFT00000018397)	deleterious low confidence	NA
4	19114717	GT	D > E/D > E	missense variant	0/0	1/1	DNAJC12 (ENSCAFT00000021116/ENSCAFT00000049485)	tolerated (0.1/0.28)	benign/possibly damaging (0.023/0.579)
4	75351270	A > G	V > A	missense variant	0/0	1/1	PDZD2 (ENSCAFT00000047348)	tolerated (0.33)	benign (0.000)
5	21123033	C > T	R > Q/R > Q/R > Q	missense variant	0/0	1/1	DIXDC1 (ENSCAFT00000022334/ENSCAFT00000022333/ENSCAFT00000035298)	tolerated (1/1/1)	benign (0.000/0.003/0.001)
5	48648175	G > C	-	non coding transcript exon variant, non coding transcript variant	0/0	1/1	ENSCAFG00000018860 (novel gene; ENSCAFT00000029943)	NA	NA
5	60223074	G > T	P > Q	missense variant	0/0	1/1	ACOT7 (ENSCAFT00000049704)	deleterious low confidence	possibly damaging (0.694)
6	36821482	G > T	S > Y	missense variant	0/0	1/1	C16orf96 (ENSCAFT00000048346)	deleterious (0)	probably damaging (0.995)
6	38937765	C > T	A > V	missense variant	0/0	1/1	ZNF598 (ENSCAFT00000030940)	tolerated (0.62)	benign (0.017)
6	40648375	C > T	P > S/P > S/P > S	missense variant	0/0	1/1	ENSCAFG00000024344 (novel gene; ENSCAFT00000037586/ENSCAFT00000037216/ENSCAFT00000037580)	deleterious (0/0/0)	probably damaging (1.00/1.00/1.00)
6	56637047	TACAA > T	F76fs	frameshift variant	0/0	1/1	RPAP2 (novel gene; ENSCAFT00000032095)	NA	NA
6	56760354	T > C	K > E	missense variant	0/0	1/1	KIAA1107 (ENSCAFT00000032101)	tolerated (0.26)	benign (0.053)
6	56786359	G > C	D > E	missense variant	0/0	1/1	KIAA1107 (ENSCAFT00000032101)	tolerated (1)	benign (0.000)
6	57204844	A > G	Y > C	missense variant	0/0	1/1	TGFBR3 (ENSCAFT00000032134)	deleterious (0)	probably damaging (0.998)
9	3074837	G > C	H > Q/H > Q/H > Q	missense variant	0/0	1/1	TNRC6C (ENSCAFT00000048770/ENSCAFT00000050041/ENSCAFT00000008443)	tolerated (0.12/0.8/0.72)	probably damaging (0.999/0.988/0.999)
11	62457942	G > A	A > T	missense variant	0/0	1/1	ZNF462 (ENSCAFT00000004432)	NA	benign (0.000)
13	19383758	G > A	E > K	missense variant	0/0	1/1	MTBP (ENSCAFT00000001477)	deleterious (0.01)	probably damaging (0.979)
13	58949521	C > G	L > V/L > V	missense variant	0/0	1/1	UGT2B31 (ENSCAFT00000022724/ENSCAFT00000004520)	tolerated (1/1)	benign (0.000/0.000)
13	58949524	G > A	V > I/V > I	missense variant	0/0	1/1	UGT2B31 (ENSCAFT00000022724/ENSCAFT00000004520)	tolerated (0.49/0.48)	benign (0.005/0.001)
13	58949536	A > G	T > A/T > A	missense variant	0/0	1/1	UGT2B31 (ENSCAFT00000022724/ENSCAFT00000004520)	tolerated (0.66/0.65)	benign (0.000/0.000)
15	42583230	A > C	N > H/N > H	missense variant	0/0	1/1	HCFC2 (ENSCAFT00000011975/ENSCAFT00000011972)	tolerated (0.09)/deleterious (0.04)	possibly damaging (0.583/0.616
17	58777715	G > A	S > L	missense variant	0/0	1/1	TXNIP (ENSCAFT00000018124)	tolerated (0.2)	benign (0.126)
18	40794747	C > T	R > H	missense variant	0/0	1/1	OR10H12 (ENSCAFT00000037956)	tolerated (0.37)	benign (0.000)
20	54717650	G > A	T > M	missense variant	0/0	1/1	KDM4B (ENSCAFT00000030040)	tolerated (0.05)	possibly damaging (0.934)
20	54729635	A > G	-	splice acceptor variant	0/0	1/1	KDM4B (ENSCAFT00000030040)	NA	NA
22	30574626	C > T	T > M	missense variant	0/0	1/1	CLN5 (ENSCAFT00000008156)	deleterious (0.01)	probably damaging (1.000)
24	46507770	C > T	R > W	missense variant	0/0	1/1	ENSCAFG00000030917 (novel gene; ENSCAFT00000046966)	NA	NA
27	31792218	T > C	I > V	missense variant	0/0	1/1	C12orf60 (ENSCAFT00000020587)	tolerated (0.39)	benign (0.009)
28	34998871	G > A	V > I	missense variant	0/0	1/1	BCCIP (ENSCAFT00000020430)	tolerated (0.24)	possibly damaging (0.992)
30	29440908	C > T	E > K	missense variant	0/0	1/1	RASL12 (ENSCAFT00000027120)	tolerated (0.08)	benign (0.034)
30	29986720	G > A	E > K	missense variant	0/0	1/1	SLC24A1 (ENSCAFT00000027314)	tolerated (0.09)	benign (0.223)
32	22124585	GTCTTT > G	K64fs	frameshift variant (microsatellite)	0/0	1/1	ENSCAFG00000031054 (novel gene; ENSCAFT00000047190)	NA	NA
37	502225	CCTTGTGCAA > C	L4_K6del	inframe deletion	0/0	1/1	OSGEPL1 (ENSCAFT00000014928)	NA	NA
38	20346455	T > A	V > D	missense variant	0/0	1/1	C1orf111 (ENSCAFT00000036623)	tolerated (0.94)	benign (0.000)

In total 37 variants with predicted high or moderate effects (SNPEff) could be exclusively found homozygous for the mutant allele in SPAID-affected Shar-Pei. SIFT and PolyPhen-2 variant effect estimations are shown for each position

**Table 3:** Case–control test results for candidate variants for SPAID

CFA	Polymorphism	MAF total	Wild type allele	Minor allele	MAF controls	MAF cases	Allele odds ratio	Chi-Square Genotype (Probability)	Chi-Square Allele (Probability)	Chi-Square Trend (Probability)
1	*CD79A*:g.112413114insGTGATG	0.149	C	del	0.137	0.152	1.128	2.287 (*P* = 0.319)	0.136 (*P* = 0.712)	0.156 (*P* = 0.693)
6	*C16orf96*:g.36821482G > T	0.149	G	C	0.169	0.142	1.230	2.445 (*P* = 0.295)	0.442 (*P* = 0.507)	0.438 (*P* = 0.508)
6	*ENSCAFG00000024344*:g.40648375C > T	0.334	C	C	0.387	0.305	0.695	4.450 (*P* = 0.108)	2.311 (*P* = 0.128)	2.470 (*P* = 0.116)
6	*RPAP2*:g.56637047delACAA	0.301	TACAA	ins	0.307	0.299	1.036	0.436 (*P* = 0.804)	0.020 (*P* = 0.887)	0.020 (*P* = 0.887)
6	*TGFBR3*:g.57204844A > G	0.455	A	G	0.436	0.465	0.886	6.180 (*P* = 0.050)	0.276 (*P* = 0.599)	0.267 (*P* = 0.605)
13	*MTBP*:g.19383758G > A	0.247	G	G	0.395	0.157	3.517	25.671 (*P* = 2.664E-06)	23.550 (*P* = 1.217E-06)	23.551 (*P* = 1.217E-06)
15	*HCFC2*:g.42583230A > C	0.408	A	C	0.395	0.422	0.896	0.193 (*P* = 0.908)	0.222 (*P* = 0.637)	0.192 (*P* = 0.661)
22	*CLN5*:g.30574626C > T	0.214	C	T	0.266	0.177	1.692	3.787 (*P* = 0.151)	3.732 (*P* = 0.053)	3.633 (*P* = 0.057)
37	*OSGEPL1*:g.502225delCTTGTGCAA	0.485	CCTTGTGCAA	del	0.500	0.466	0.872	1.341 (*P* = 0.511)	0.364 (*P* = 0.546)	0.388 (*P* = 0.533)

SPAID-associated variants with predicted high or moderate effects (SNPEff) were genotyped for 102 SPAID-affected and 62 SPAID-unaffected Shar-Pei

### Validation of genetic variants

Validation of all nine potentially deleterious genetic variants detected in whole genome sequencing data in 102 SPAID-affected and 62 SPAID-unaffected Shar-Pei using Kompetitive Allele Specific PCR (KASP) and gel electrophoresis revealed a borderline significant P-value for *TGFBR3*:g.57204844A > G located in *TGFBR3 (transforming growth factor beta receptor 3;* P = 0.050), but a highly significant P-value for *MTBP*:g.19383758G > A (P = 2.664E-06) located in *MTBP (Mdm2, transformed 3 T3 cell double minute 2, p53 binding protein*). Further validation of all nine variants in 162 dogs of 11 different breeds showed that none of the variants could be found in any other tested dog breed (Additional file 3: Table S3). The missense mutation *MTBP*:g.19383758G > A was predicted to result in a deleterious (0.01; SIFT) as well as probably damaging (0.979; PolyPhen-2) substitution of glutamic acid to lysine located in the MDN2-binding protein, C-terminal domain.

Genotypic distribution of *MTBP*:g.19383758G > A in Shar-Pei of all age groups (1–14 years) revealed none of the investigated SPAID-affected Shar-Pei harboring a homozygous wild type genotype. In total, 68.63% showed the A/A genotype whereas 31.37% had a heterozygous genotype (G/A). In the validated SPAID-unaffected Shar-Pei we found the G/G genotype in 16.13%, G/A in 45.16% and A/A in 38.71%. Further investigation of the genotypes in a subgroup of Shar-Pei older than six years revealed the disease associated genotype A/A exclusively in SPAID-affected dogs as well as the wild type genotype G/G only in SPAID-unaffected Shar-Pei (Table 4). In 25 Shar-Pei with a heterozygous genotype, 32% were not affected at the date of examination whereas 68% revealed signs of SPAID and therefore showed a 2.13-fold higher risk for disease development. The distribution of genotypes by phenotypes suggested an incomplete dominant mode of inheritance. Complex segregation analysis supported this mode of inheritance because a significant difference based on a log-likelihood ratio test (differences between −2 log-likelihoods) among a model with arbitrary Mendelian factors and a model accounting for environmental variation (*χ* ^2^ = 11.58, df = 5, p = 0.040) could be found, whereas other models (recessive or polygenic models) did not result in significant differences. With regard to the breed specific types, the modern type with a meatmouth was found to be most frequent among SPAID-affected Shar-Pei (83.3%) although all three genotypes were detected in meatmouth as well as bonemouth Shar-Pei.

**Table 4:** Genotypic distribution of SPAID-associated missense mutation *MTBP*:g.19383758G > A

Phenotype	Number of Shar Pei (n)	Genotype G/G	Genotype G/A	Genotype A/A
Age group (>6 years)				
SPAID affected Shar-Pei	46	0	17	29
SPAID unaffected Shar-Pei	11	3	8	0
All age groups (1–14 years)				
SPAID affected Shar-Pei	102	0	32	70
SPAID unaffected Shar-Pei	62	10	28	24
Muzzle types (all age groups)				
meatmouth	132	3	41	88
bonemouth	32	7	19	6

None of the affected Shar-Pei older than six years showed the wild type genotype G/G whereas none of the unaffected Shar-Pei of this group harboured the disease associated genotype A/A. Regardless of the muzzle type both genotypes G/G or A/A could be found in meatmouth and bonemouth Shar-Pei types

### ddPCR

*MTBP*:g.19383758G > A was located 1 Mb proximal to the copy number variation CNV_16.1 on CFA13. Further detection of CNV_16.1 by ddPCR revealed stable alleles of one or five copies. These copy numbers were in perfect cosegregation with the genotypes of *MTBP*:g.19383758G > A (Table 5).

**Table 5:** Comparison of *MTBP*:g.19383758G > A genotypes with CNV_16.1

*MTBP*:g.19383758G > A	A/A	A/G	G/G
All age groups (1–14 years)			
SPAID affected Shar-Pei	17	3	0
SPAID unaffected Shar-Pei	3	6	2
CNV mean (RT-PCR)	10.05	5.22	2.0
CNV (ddPCR)	10	6	2
*MTBP*:g.19383758G > A	A/A	A/G	G/G
Age group >6 years			
SPAID affected Shar-Pei	8	3	0
SPAID unaffected Shar-Pei	0	3	1
CNV mean (RT-PCR)	9.5	5.0	2.0
CNV (ddPCR)	10	6	2

The results of CNV detection by digital droplet PCR show perfect cosegregation with *MTBP*:g.19383758G > A

### Candidate gene sequencing

Sanger sequencing results of *MTBP* (ENSCAFT00000001477) in one SPAID-affected Shar-Pei and two control dogs confirmed the candidate SNV *MTBP*:g.19383758G > A (ss2136554981) in the complementary DNA in the affected Shar-Pei (Additional file 4: Table S4) and the *MTBP* gene model predicted by Ensembl. In addition, we detected four SNVs which could not be exclusively found in the affected Shar-Pei.

### Expression analysis

To investigate the potential functional effect of *MTBP*:g.19383758G > A variant on MTBP we studied *TP53 (cellular tumor antigen p53)* mRNA expression as a bioindicator for MTBP. MTBP was shown to form a complex with MDM2 and thereby stabilizes this protein [24]. MDM2 is an inhibitor of p53, encoded by *TP53* [25]. Thus, MTBP indirectly inhibits p53 by stabilizing MDM2 in the p53-MDM2-MTBP pathway [25].

**Fig. 5:**
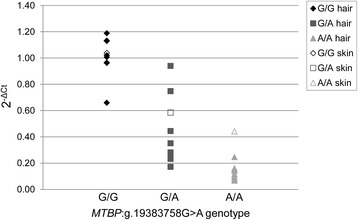
Relative expression levels of *TP53*. Relative expression of *TP53* and its relation to *MTBP*:g.19383758G > A genotypes are shown. The ΔΔCT method was used to compute expression levels (2^-ΔCt^) for skin and hair tissues. Compared to samples with a homozygous wild type genotype (G/G), the relative expression levels of samples with a heterozygous genotype (G/A; p < 0.0001) or homozygous mutant genotype (A/A; p < 0.0001) were significantly decreased

Quantification of *TP53* levels performed in skin and hair samples of all three *MTBP*:g.19383758G > A genotypes G/G, G/A and A/A revealed a significant decrease of relative *TP53* expression in dogs with a heterozygous genotype (*p* < 0.0001) as well as in dogs with a homozygous mutant genotype (*p* < 0.0001) in relation to the wild type genotype (Fig. 5). In-between G/A and A/A genotypes the differential expression was also significant (*p* = 0.0034). It could be observed that relative expression levels of G/A-samples showed the highest variation ranging from low 2^-ΔCt^ (min 2^-ΔCt^ = 0.23), which could also be found in A/A samples, to higher 2^-ΔCt^ (max 2^-ΔCt^ = 0.94) found in G/G samples. Skin samples showed a similar distribution of relative expression levels and confirmed a significant decrease in G/A and an even higher decrease in A/A samples.

### Runs of homozygosity (ROH)

Analysis for ROH regions was performed to investigate homozygosity in the region of *MTBP* and to identify further potential signatures of selection in the same SPAID-affected Shar-Pei which were studied in whole genome sequencing analysis. In total, 12 shared ROH regions could be detected in canine high density bead chip data of the two Shar-Pei, which could not be found in the investigated eight control dogs of different breeds (Additional file 5: Table S5). ROHs were identified on CFA6, 13, 18, 19, 22, 30, 35, 36 and 38. The largest ROH region was found on CFA13 at 18,625,926-19,967,354 bp (CanFam3.1) including *MTBP* as well as *ENPP2 (ectonucleotide pyrophosphatase/phosphodiesterase 2), TAF2 (TATA-box binding protein associated factor 2), DSCC1 (DNA replication and sister chromatid cohesion 1), DEPTOR (DEP domain containing MTOR-interacting protein), COL14A1 (collagen type XIV alpha 1 chain), MRPL13 (mitochondrial ribosomal protein L13), SNTB1 (syntrophin beta 1), SYT17 (synaptotagmin 17), RNA5SP34 (RNA, 5S ribosomal pseudogene 34)* and seven novel genes. On CFA6 only one novel gene (ENSCAFT00000051190) could be identified in the ROH region as well as the three genes *CNN3 (calponin 3), TF (transferrin)* and *ABCD3 (ATP binding cassette subfamily D member 3)* in close proximity to this region.

Functional annotation for human orthologue genes detected in all ROH regions found exclusively in Shar-Pei showed a particularly high number of genes involved in cellular processes (GO:0009987) and metabolic processes (GO:0008152; Additional file 6: Table S6). In pathway analysis an inflammation mediated by chemokine and cytokine signalling pathway (P00031) was detected for the gene *COL14A1.* Nevertheless, no SPAID-associated variant with predicted high or moderate effect could be found in this gene and further candidate genes from ROH analysis. Further pathways were transcription regulator (P00023/P00055), metabolic (P02782), ubiquitin proteasome (P00060) and integrin signalling pathways (P00034).

## Discussion

Analysis of NGS data in two SPAID-affected Shar-Pei and further validation revealed *MTBP*:g.19383758G > A on CFA13 to be highly associated with SPAID in Shar-Pei. All 102 SPAID-affected dogs harbored at least one mutant A-allele of the missense mutation predicted to have a damaging effect. In SPAID-unaffected Shar-Pei 24 dogs were found to harbor the homozygous mutant genotype (A/A). These dogs were all younger than six years, whereas none of the older dogs showed the A/A genotype. We suppose that due to the variable onset of the disease SPAID-unaffected Shar-Pei with homozygous mutant genotype, which are younger than six years, might still develop signs of the disease. Shar-Pei at an age of at least seven years with no signs of the disease were proposed to be most likely healthy with regard to SPAID [14].

Under the assumption of a variable age of onset, the results propose an incomplete dominant mode of inheritance for *MTBP*:g.19383758G > A. This suggestion was supported by the high incidence of SPAID-affected Shar-Pei as much as by the identification of a large ROH region exclusively found in two SPAID-affected Shar-Pei but not in control dogs. ROH detection has been shown to be a successful method for the investigation of disease traits as well as other phenotypic traits [12, 26–29]. Long ROHs were reported to be frequent in genomic regions harboring genes associated with autosomal-dominant diseases [30]. Analysis for signatures of selection specific for Shar-Pei gave evidence for a region on CFA13 harboring *HAS2,* which was suggested to be associated with breed specific skin wrinkling [12]. Further genome screening for signatures of selection in 50 US-Shar-Pei detected a potential signature of selective sweeps downstream of *HAS2* and revealed further reduction of homozygosity on CFA5, 6, 13 and X [11]. The results from present ROH analysis in two SPAID-affected Shar-Pei genotyped on the canine Illumina high density bead chip identified a ROH region proximal of the previously detected ROHs harboring the candidate gene *MTBP*. Possible reasons for this shift of ROH regions might be the differences in marker density and investigated populations (US and German Shar-Pei) in these studies but might also be the specific testing for SPAID-affected Shar-Pei in our analysis. We assume that the gene locus for a thick and wrinkled skin responsible for the meatmouth Shar-Pei type might be close to the causative variant for SPAID and could explain the assumption that CNV_16.1 near *HAS2* is related to affected skin formation simultaneously to periodic fever [11]. Despite the limitation of our analysis due to the low number of tested Shar-Pei, the detection of a highly SPAID-associated variant in *MTBP* supports this assumption.

The main role of MTBP was shown to be the formation of a stable complex with MDM2 by preventing it from autoubiquitination [24, 25]. MDM2 could be shown to have a pro-inflammatory as well as an epithelial regeneration effect [31]. A blockade of MDM2 investigated in postischemic kidneys resulted in a suppression of a sterile inflammatory response, an enhanced apoptosis and intrarenal expression of proapoptotic p53 target genes [31]. A similar suggestion was made for variants in *MFEV* resulting in human Mediterranean Fever which did not only increase sensitivity for triggers of inflammatory responses but did also play a role in apoptotic processes [32]. In addition, MTBP was proposed to promote indirectly the degradation of p53 by MDM2-mediated ubiquitination [25]. Due to this strong relationship in the MTBP-MDM2-p53 pathway, *TP53* mRNA was used a bioindicator for MTBP-MDM2 activity. It was shown that an increase of MDM2 leads to a decrease of p53 levels and finally also a decrease of MDM2 in a feedback loop [24]. In our analysis for SPAID we detected this effect as well, showing a decrease of *TP53* expression in Shar-Pei with a mutant G/A or A/A genotype due to the p53-inhibitory function of MDM2. For this reason, we assume that a modification in the MDM2-binding domain of MTPB protein in SPAID-affected dogs might affect the binding affinity to MDM2 and potentially result in a higher complex stability or reduced complex dissociation [24] and consequently increase MDM2 activity. Due to this functional relation and the distribution of the mutant allele in tested dogs, we assume that the missense mutation *MTBP*:g.19383758G > A induces proinflammatory effects via MTBP-MDM2 pathway in SPAID-affected Shar-Pei.

This variant is located near CNV_16.1 which has previously been suggested to increase SPAID-risk in Shar-Pei dogs [15]. In our analyses this CNV showed complete linkage with *MTBP*:g.19383758G > A. Nevertheless, it was suggested that CNV_16.1 might alter the expression of *HAS2* and thereby affect both the development of a meatmouth muzzle type and SPAID [11]. The distribution of genotypes actually confirmed the high incidence of SPAID in meatmouth Shar-Pei types but also detected SPAID-affected bonemouth types. In addition, *HAS2* mRNA was further shown to be highly expressed in cells of severely folded skin and skin vesicles derived from vesicular hyaluronosis due to an accumulation of hyaluronic acid [33, 34]. Our pathohistologic examinations showed that both SPAID-affected and SPAID-unaffected dogs harboured signs of cutaneous mucinosis as a result of hyaluronic acid accumulation. For this reason we propose that hyaluronic acid levels are not related to SPAID. Thereby, the role of CNV_16.1 and the influence of HAS2 on SPAID remain unclear. In contrast, mutant MTBP is not related to hyaluronic acid accumulation but to inflammatory and apoptotic processes via MDM2 and thus suggests an important role in SPAID development. Nevertheless, due to the complex nature of inflammatory processes we cannot exclude that further variants might play a role in disease development.

Functional analysis of genes detected in potential signatures of selection in SPAID-affected Shar-Pei revealed a high number of genes involved in signaling pathways that might be involved in inflammatory processes. Investigations of inflammatory mediators in alveolar epithelial derived from inflamed lungs in mice proposed that especially genes involved in signal transduction were downregulated in cells affected by inflammation [35]. Particularly *COL14A1n* which could be found to be involved in the inflammation mediated by chemokine and cytokine signaling pathway in our ROH region analysis, was also reported to be downregulated in inflamed lung cells in mice [35]. The candidate gene *DEPTOR* was identified as a further proinflammatory gene in this ROH region on CFA13, involved in the regulation of vascular endothelial cell activation and proinflammatory responses [36]. In addition, ABCD3 encoding for a peroxisomeal membrane protein was detected close to a ROH region on CFA6. It was shown that ABCD3 was involved in the protection of cortical neurons from inflammatory mediators [37]. Although no SPAID-associated variants with predicted high or moderate effects could be detected in these genes, it cannot be excluded that further functional variants might affect them and might therefore be involved in the autoinflammatory processes in SPAID-affected Shar-Pei.

## Conclusions

In conclusion, in this study we showed a missense variant in *MTBP* acting via MDM2 for a proinflammatory reaction in SPAID-affected Shar-Pei. These results represent an important advance in the efforts to elucidate genetic mechanisms involved in pre-inflammatory processes in dogs.

## Methods

### Animals

The genomic DNA of 164 Shar-Pei dogs and 162 dogs from different breeds as controls was isolated from EDTA-blood samples and adjusted to 10 ng/μl. The health status of all Shar-Pei dogs was determined by clinical examination by the authors (28 Shar-Pei), accredited veterinarians and the owners observations recorded in a questionnaire (Additional file 7: Table S7).

In addition, in 132 study participants a supplementary form was filled in by dog owners and their veterinarians to specify the types of inflammatory processes. Specific parameters like the occurrence of fever, arthritis, erythema, thickened and pasty skin, otitis, eye inflammation and recurrent intestinal inflammation were assessed in this form. Two of SPAID-affected Shar-Pei with severe clinical signs including recurrent bouts of fever and arthritis were chosen for whole-genome sequencing.

### Histopathologic examination

In two SPAID-affected and one SPAID-unaffected Shar-Pei a full necropsy was carried out. Tissue samples from skin, mamma, lymph nodes (pulmonales and mesenteriales), tonsils, salivary gland, liver, pancreas, kidneys, adrenal glands, parathyroid glands, thyroid glands, spleen, lamina propria mucosae of stomach, small and large intestine were fixed in 10% formalin and embedded in paraffin wax. Paraffin sections (4 μm) were stained with hematoxylin and eosin (HE) for histology. Mucin was stained applying the combined alcian blue (pH 2.5)/periodic acid Schiff reaction (AB/PAS). For detection of amyloid, sections of all organs were stained with Congo red and examined using light microscopy. Presence of amyloid was confirmed by examination of Congo red-stained sections under polarized light. Photographs were taken with a light microscope (Olympus BX51, Olympus, Hamburg, Germany) with an Olympus camera DP72 and Olympus cellSense software.

### Whole-genome sequencing

For library preparation, genomic DNA of two Shar-Pei affected with recurrent bouts of fever was isolated from blood samples using Invisorb Spin Blood Mini Kit (STRATEC biomedical, Birkenfeld, Germany). DNA samples were enzymatically tagmented and fragmented using Nextera Library Preparation Kit (Illumina, San Diego, CA) and quality controlled on the Bioanalyzer High Sensitivity DNA Kit (Agilent, Santa Clara, California). NGS was performed on the Illumina MiSeq for 2x300 bp reads in paired-end mode. In total two runs with v3 Reagent Kits (15 Gb, Illumina) were carried out for each SPAID-affected Shar-Pei (BioProject PRJNA327712). We performed quality control using fastqc 0.11.5 [38], mapping to the reference genome CanFam 3.1 (Ensembl) using BWA 0.7.13 [39] and variant calling using SAMtools 1.3.1 [40], Picard tools (http://broadinstitute.github.io/picard/, version 2.3.0) and GATK 3.5 [41]. Five fastq files from a Korean Jindo Dog (DRR001566), an Afghan Hound (SRR1061643), two German Shepherd dogs (SRR1130247, SRR1124304) and a Border Collie (SRR654728) derived from Sequence Read Archive (NCBI) were included in this study as controls. Variants with a read depth of 3–999 and quality values >20 were chosen for further analysis and investigated for their potential effects using SNPEff version 4.1 g [42].

### Selection of variants

Variants derived from whole genome sequencing analysis were filtered for homozygous mutant genotypes exclusively found in SPAID-affected Shar-Pei with predicted high or moderate effects using SAS, version 9.4 (Statistical Analysis System, Cary, NC). Variants which could be found in the dbSNP database assigned as known variants or which were predicted to be located in an incomplete region of the reference genome (incomplete transcript) were omitted from analysis. The remaining variants were investigated for their potential effects on the protein using SIFT [43] and PolyPhen-2 [44]. Those SNVs which were predicted to be deleterious by SIFT and also predicted to be probably damaging by PolyPhen-2 as well as three INDEL were chosen for validation.

### Validation

All nine variants were genotyped in all 102 SPAID-affected, 62 SPAID-unaffected Shar-Pei and 162 dogs of 11 different breeds. For six SNVs KASP assays (LGC Genomics, Middlesex, UK; [45]) were designed and run on an ABI7300 real-time system for 96 well plates (Additional file 8: Table S8). Further three variants were genotyped using gel electrophoresis on an acrylamide gel and a LI-COR 4300 DNA Analyzer (LI-COR, Lincoln, Nebraska). Allele and genotype distributions and their association with SPAID were investigated applying the CASECONTROL procedure from SAS.

### Sanger sequencing

Skin tissues for RNA isolation were available from an SPAID-affected Shar-Pei that was euthanized due to signs of severe renal dysfunction including sickness, diarrhea, fever and swollen joints. In addition skin tissues from an Irish Wolfhound and English Springer Spaniel which were euthanized due to orthopedic problems were used as reference samples. RNA was extracted according to standard protocols for the RNeasy Mini Kit (QIAGEN, MD, USA). Tissues were homogenized in QIAzol Lysis Reagent (QIAGEN) using the Precellys homogenizer (Bertin Technologies, Montigny le Bretonneux, France). The obtained RNA was transcribed into cDNA using the Maxima First Strand cDNA Synthesis Kit (Thermo Fisher Scientific, Fermentas, Waltham, MA, USA). For Sanger sequencing primers (Additional file 9: Table S9) were designed using Primer3 software (version 0.4.0, http://bioinfo.ut.ee/primer3-0.4.0/) and PCR reactions were run on a thermocycler TProfessional 96 (Biometra, Göttingen, Germany).

### Segregation analysis

Complex segregation analysis was performed for five pedigrees with 79 individuals using the procedure SEGREG of S.A.G.E. (Statistical Analysis for Genetic Epidemiology, release 6.4, http://darwin.cwru.edu). A regressive logistic model was used to test for the most probable type of inheritance with the age of onset as covariate.

### ddPCR

Detection of CNV_16.1 was performed in 31 Shar-Pei using QX200 Droplet Digital PCR (ddPCR) System (BIO RAD, Munich, Germany). Primers, probes and reference gene were used according to previous analysis [11]. The DNA was digested using HaeIII restriction enzyme (New England Bio Labs, Ipswich, MA).

### Expression analysis

RNA was isolated from skin and hair samples and transcribed into complementary DNA (cDNA) according to standard protocols [46]. In total, two skin samples from Great Danes with *MTBP*:g.19383758G > A genotype G/G and two samples from SPAID-affected Shar-Pei with a G/A and a A/A genotype were used for analysis. Hair root samples were derived from nine SPAID-affected Shar-Pei with a homozygous mutant genotype (A/A), nine Shar-Pei with a heterozygous genotype (G/A; four dogs with signs of the disease and five dogs without signs of the disease) and further seven dogs with a G/G genotype including one Shar-Pei and six Great Danes as controls. A FAM-labeled custom assay for *RPL13A* (primer forward: GCCAAGATCCATTATCGGAAGAAGA, primer reverse: CCACATTCTTTTCGGCCTGTTT, reporter: CCGTAGCCTCATAAGCT) as housekeeping gene, previously shown to be one of the most stably expressed genes in canine whole skin [47], a VIC-labeled gene expression assay for *TP53* (ID: Cf02623149_m1, reporter: GAAAACAATGTTCTGTCTTCGGAGC) and TaqMan gene expression master mix (Thermo Fisher Scientific) were used for quantitative real-time (qRT)-PCR on an ABI7300 sequence detection system (Thermo Fisher Scientific, Applied Biosystems instrument). All samples were analyzed in triplicates. Relative expression levels were calculated using the ΔΔCT method [48]. Mean ΔCT of dogs with G/G genotype was assigned as reference.

### ROH detection

Two SPAID-affected Shar-Pei and eight control dogs from different breeds (Bernese Mountain dog, Dalmatian, German shepherd, Lagotto Romagnolo, Parson Russell Terrier, Samoyed, Boxer, German Pinscher) were genotyped on the canine Illumina high density bead chip (Illumina) according to manufacturer’s protocols. A genotyping rate per sample >0.70 and a genotyping rate per SNP of >0.90 were applied for quality control and resulted in 157,642 SNPs for ROH analysis. ROHs were detected in sliding windows of 10 SNPs with homozygous regions of >120 kb using PLINK, version 1.90 (www.cog-genomics.org/plink/1.9/). A maximum of two SNPs with missing genotypes and no heterozygous SNPs were admitted in each window. Consensus ROHs regions detected in both Shar-Pei which could not be found in control dogs were investigated for human orthologues using Galaxy intersection tool (https://usegalaxy.org/) [49, 50], g:Profiler [51] and PANTHER gene list analysis for biological processes and pathways (release 2016-07-15, version 11.1, [52]).

## Additional files

Additional file 1: Table S1. Phenotypic data of 164 study participants with specified inflammatory process types. The number of Shar-Pei dogs and their individual signs of inflammation is shown (1 = affected; 0 = unaffected). In 62 Shar-Pei no signs of the disease could be found. (PDF 260 kb)

Additional file 2: Table S2. Whole genome sequencing statistics for two SPAID-affected Shar-Pei and five controls. DNA-libraries of two Shar-Pei were sequenced on the Illumina MiSeq whereas further five whole genome sequences from dogs of four different breeds were derived from sequence read archive (NCBI). (DOCX 15 kb)

Additional file 3: Table S3. Mutant allele frequencies for SPAID candidate variants. All nine variants were investigated for their genotypic distribution in SPAID-affected and unaffected Shar-Pei as well as in 162 dogs of 11 different breeds. (DOCX 20 kb)

Additional file 4: Table S4. Variant detection in complementary DNA of *MTBP*. In total five variants could be detected in cDNA of *MTBP* including the candidate SNV *MTBP*:g.19383758G > A. None of the other variants could be exclusively found in the SPAID-affected Shar-Pei. (DOCX 17 kb)

Additional file 5: Table S5. Table S2. Shared runs of homozygosity (ROH) for SPAID-affected Shar-Pei. Two Shar-Pei were investigated for shared ROH regions which could not be detected in eight control dogs of different breeds. The chromosomal position (CanFam2.0 and CanFam3.1) of shared ROH regions, size of shared ROHs, number of SNPs, genes IDs (CanFam3.1) and human orthologues are shown. (DOCX 20 kb)

Additional file 6: Table S6. The proportion of gene hits against total number of process hits for genes detected in runs of homozygosity (ROH) regions shared by SPAID (Shar-Pei Autoinflammatory Disease) affected Shar-Pei, which could not be found in control dogs, is shown. PANTHER gene list analysis for biologic processes and pathways was done for human orthologues. (DOCX 16 kb)

Additional file 7: Table S7. Questionnaire for the assessment of health status in investigated Shar-Pei. A description of the project and information consent for dog owners and veterinarians is included. (PDF 160 kb)

Additional file 8: Table S8. Primer sequences and assays for the validation of candidate variants. Kompetitive Allele Specific PCR (KASP) assays were used for six variants whereas further three variants were genotyped by gel electrophoresis on an acrylamide gel. Primer pairs, validation type, amplicon size, annealing temperature and number of PCR cycles are shown. (DOCX 17 kb)

Additional file 9: Table S9. Primers for sequencing MTBP complementary DNA. The gene regions, product sizes and annealing temperatures are shown (DOCX 14 kb)

## Abbreviations

AB: Alcian blue
ADCY8: Adenylate Cyclase 8
CNV: Copy number variation
COL14A1: Collagen Type XIV Alpha 1 Chain
ddPCR: Droplet digital PCR
DEPTOR: Domain-Containing Protein 6
DSCC1: DNA Replication And Sister Chromatid Cohesion 1
ENPP2: Ectonucleotide Pyrophosphatase/Phosphodiesterase 2
HAS2: Hyaluronan synthase 2
HE: Hematoxylin and eosin
MEFV: Mediterranean Fever
MRPL13: Mitochondrial Ribosomal Protein L13
MTBP: MDM2 Binding Protein
PAS: Periodic acid Schiff reaction
SNTB1: Syntrophin Beta 1
SPAID: Shar-Pei Autoinflammatory Disease
SRA: Sequence read achieve
TAF2: TATA-Box Binding Protein Associated Factor 2
