# Supplementary material for: Whole genome sequencing identifies missense mutation in MTBP in Shar-Pei affected with Autoinflammatory Disease (SPAID)
Source: BMC Genomics. 2017 May 4;18:348. doi: 10.1186/s12864-017-3737-z (PMC5418765; doi:10.1186/s12864-017-3737-z)
Supplement: Supplementary file 2 — Whole genome sequencing statistics for two SPAID-affected Shar-Pei and five controls. DNA-libraries of two Shar-Pei were sequenced on the Illumina MiSeq whereas further five whole genome sequences from dogs of four different breeds were derived from sequence read archive (NCBI). (DOCX 15 kb) [file 12864_2017_3737_MOESM2_ESM.docx]

**Table S2. Whole genome sequencing statistics for two SPAID-affected Shar-Pei and five controls.** DNA-libraries of two Shar-Pei were sequenced on the Illumina MiSeq whereas further five whole genome sequences from dogs of four different breeds were derived from sequence read archive (NCBI).

|  | Platform | Number of lanes | Average read length | Bases mapped | Mean coverage | Number of SNPs | Number of INDELs |
| --- | --- | --- | --- | --- | --- | --- | --- |
| SPAID-affected Shar-Pei 1 | Illumina MiSeq | 2 | 221 | 25786454419 | 11.08 | 13978236 | 3299267 |
| SPAID-affected Shar-Pei 2 | Illumina MiSeq | 2 | 287 | 27757561652 | 11.93 | 14365345 | 3623708 |
| Korean Jindo Dog (DRR001566) | Illumina HiSeq 2000 | 1 | 100 | 104524095864 | 45.35 | 10750681 | 3251857 |
| Afghan Hound (SRR1061643) | Illumina HiSeq 2000 | 1 | 101 | 18219774542 | 9.63 | 10215977 | 2913055 |
| German Shepherd (SRR1130247) | Illumina HiSeq 2000 | 1 | 100 | 38338850458 | 18.79 | 10626117 | 3197109 |
| German Shepherd (SRR1124304) | Illumina HiSeq 2000 | 1 | 100 | 38533923645 | 18.88 | 10635537 | 3206394 |
| Border Collie (SRR654728) | Illumina HiSeq 2000 | 1 | 101 | 25280414492 | 12.54 | 10590998 | 3186690 |
