# Supplementary material for: Whole genome sequencing identifies missense mutation in MTBP in Shar-Pei affected with Autoinflammatory Disease (SPAID)
Source: BMC Genomics. 2017 May 4;18:348. doi: 10.1186/s12864-017-3737-z (PMC5418765; doi:10.1186/s12864-017-3737-z)
Supplement: Supplementary file 3 — Mutant allele frequencies for SPAID candidate variants. All nine variants were investigated for their genotypic distribution in SPAID-affected and unaffected Shar-Pei as well as in 162 dogs of 11 different breeds. (DOCX 20 kb) [file 12864_2017_3737_MOESM3_ESM.docx]

**Table S3. Mutant allele frequencies for SPAID candidate variants.** All nine variants were investigated for their genotypic distribution in SPAID-affected and unaffected Shar-Pei as well as in 162 dogs of 11 different breeds.

| CFA | 1 | 6 | 6 | 6 | 6 | 13 | 15 | 22 | 37 |
| --- | --- | --- | --- | --- | --- | --- | --- | --- | --- |
| Position | 112413114 | 36821482 | 40648375 | 56637047 | 57204844 | 19383758 | 42583230 | 30574626 | 502225 |
| Base change | C>CGTGATG | G>T | C>T | TACAA>T | A>G | G>A | A>C | C>T | CCTTGTGCAA>C |
| Gene | CD79A | C16orf96 | ENS-  CAFG  00000024344 | RPAP2 | TGFBR3 | MTBP | HCFC2 | CLN5 | OSGEPL1 |
| Dogs (n) | Mutant allele frequency | Mutant allele frequency | Mutant allele frequency | Mutant allele frequency | Mutant allele frequency | Mutant allele frequency | Mutant allele frequency | Mutant allele frequency | Mutant allele frequency |
| SPAID-affected Shar-Pei (102) | 0.85 | 0.86 | 0.68 | 0.70 | 0.42 | 0.84 | 0.42 | 0.18 | 0.47 |
| SPAID-unaffected Shar-Pei (62) | 0.86 | 0.83 | 0.61 | 0.69 | 0.44 | 0.60 | 0.40 | 0.27 | 0.50 |
| Appenzeller Sennenhund (16) | 0.00 | 0.00 | 0.00 | 0.00 | 0.00 | 0.00 | 0.00 | 0.00 | 0.00 |
| Bernese Mountain Dog (16) | 0.00 | 0.00 | 0.00 | 0.00 | 0.00 | 0.00 | 0.00 | 0.00 | 0.00 |
| Dachshund (15) | 0.00 | 0.00 | 0.00 | 0.00 | 0.00 | 0.00 | 0.00 | 0.00 | 0.00 |
| German Wirehaired Pointer (15) | 0.00 | 0.00 | 0.00 | 0.00 | 0.00 | 0.00 | 0.00 | 0.00 | 0.00 |
| Great Dane (16) | 0.00 | 0.00 | 0.00 | 0.00 | 0.00 | 0.00 | 0.00 | 0.00 | 0.00 |

**Table S3 continued.**

| CFA | 1 | 6 | 6 | 6 | 6 | 13 | 15 | 22 | 37 |
| --- | --- | --- | --- | --- | --- | --- | --- | --- | --- |
| Position | 112413114 | 36821482 | 40648375 | 56637047 | 57204844 | 19383758 | 42583230 | 30574626 | 502225 |
| Base change | C>CGTGATG | G>T | C>T | TACAA>T | A>G | G>A | A>C | C>T | CCTTGTGCAA>C |
| Gene | CD79A | C16orf96 | ENS-  CAFG  00000024344 | RPAP2 | TGFBR3 | MTBP | HCFC2 | CLN5 | OSGEPL1 |
| Dogs (n) | Mutant allele frequency | Mutant allele frequency | Mutant allele frequency | Mutant allele frequency | Mutant allele frequency | Mutant allele frequency | Mutant allele frequency | Mutant allele frequency | Mutant allele frequency |
| German Pinscher (8) | 0.00 | 0.00 | 0.00 | 0.00 | 0.00 | 0.00 | 0.00 | 0.00 | 0.00 |
| German Shepherd (16) | 0.00 | 0.00 | 0.00 | 0.00 | 0.00 | 0.00 | 0.00 | 0.00 | 0.00 |
| Doberman Pinscher (16) | 0.00 | 0.00 | 0.00 | 0.00 | 0.00 | 0.00 | 0.00 | 0.00 | 0.00 |
| Entlebucher Mountain Dog (16) | 0.00 | 0.00 | 0.00 | 0.00 | 0.00 | 0.00 | 0.00 | 0.00 | 0.00 |
| Greater Swiss Mountain Dog (15) | 0.00 | 0.00 | 0.00 | 0.00 | 0.00 | 0.00 | 0.00 | 0.00 | 0.00 |
| Tibetan Terrier (13) | 0.00 | 0.00 | 0.00 | 0.00 | 0.00 | 0.00 | 0.00 | 0.00 | 0.00 |
