# Supplementary material for: Whole genome sequencing identifies missense mutation in MTBP in Shar-Pei affected with Autoinflammatory Disease (SPAID)
Source: BMC Genomics. 2017 May 4;18:348. doi: 10.1186/s12864-017-3737-z (PMC5418765; doi:10.1186/s12864-017-3737-z)
Supplement: Supplementary file 4 — Variant detection in complementary DNA of MTBP. In total five variants could be detected in cDNA of MTBP including the candidate SNV MTBP:g.19383758G > A. None of the other variants could be exclusively found in the SPAID-affected Shar-Pei. (DOCX 17 kb) [file 12864_2017_3737_MOESM4_ESM.docx]

**Table S4. Variant detection in complementary DNA of *MTBP*.** In total five variants could be detected in cDNA of *MTBP* including the candidate SNV *MTBP*:g.19383758G>A. None of the other variants could be exclusively found in the SPAID-affected Shar-Pei.

| CFA | Gene | Polymorphism name | ID | Type | Source | Genotype  Shar-Pei | Genotype reference dog 1 | Genotype reference dog 2 |
| --- | --- | --- | --- | --- | --- | --- | --- | --- |
| 13 | *MTBP* | *MTBP*:g. 19321034T>C | rs22230397 | synonymous,  splice region variant | exon 5 | T/T | C/C | T/C |
| 13 | *MTBP* | *MTBP*:g.19323997T>C | rs22295780 | synonymous | exon 7 | C/C | C/C | C/C |
| 13 | *MTBP* | *MTBP*:g.19327603G>A | rs852257732 | missense variant (R>H) | exon 10 | A/A | G/G | G/A |
| 13 | *MTBP* | *MTBP*:g.19350606G>A | rs22234472 | synonymous | exon 13 | A/A | A/A | A/A |
| 13 | *MTBP* | *MTBP*:g.19383758G>A |  | missense variant (E>K) | exon 21 | A/A | G/G | G/G |
