# Supplementary material for: Whole genome sequencing identifies missense mutation in MTBP in Shar-Pei affected with Autoinflammatory Disease (SPAID)
Source: BMC Genomics. 2017 May 4;18:348. doi: 10.1186/s12864-017-3737-z (PMC5418765; doi:10.1186/s12864-017-3737-z)
Supplement: Supplementary file 5 — Table S2. Shared runs of homozygosity (ROH) for SPAID-affected Shar-Pei. Two Shar-Pei were investigated for shared ROH regions which could not be detected in eight control dogs of different breeds. The chromosomal position (CanFam2.0 and CanFam3.1) of shared ROH regions, size of shared ROHs, number of SNPs, genes IDs (CanFam3.1) and human orthologues are shown. (DOCX 20 kb) [file 12864_2017_3737_MOESM5_ESM.docx]

**Table S5. Shared runs of homozygosity (ROH) for SPAID-affected Shar-Pei.** Two Shar-Pei were investigated for shared ROH regions which could not be detected in eight control dogs of different breeds. The chromosomal position (CanFam2.0 and CanFam3.1) of shared ROH regions, size of shared ROHs, number of SNPs, genes IDs (CanFam3.1) and human orthologues are shown.

| CFA | Position (CanFam2.0) | Position (CanFam3.1) | Size of shared ROH (bp) | Number of SNPs in shared parts of ROH regions | Gene ID | Human orthologue |
| --- | --- | --- | --- | --- | --- | --- |
| 6 | 57590389-57640734 | 54535746-54586091 | 50345 | 5 | ENSCAFT00000051190.1 | N/A |
| 13 | 19274344-19824023 | 16241183-16791045 | 549679 | 13 | ENSCAFT00000001286.4  ENSCAFT00000044004.1  ENSCAFT00000046067.1  ENSCAFT00000001287.3 | RAD21  N/A  AARD  SLC30A8 |
| 13 | 20447630-20462315 | 17414728-17429421 | 14685 | 2 | ENSCAFT00000001299.4  ENSCAFT00000001302.3 | EXT1  EXT1 |
| 13 | 21658633-23004428 | 18625926-19967354 | 1345795 | 106 | ENSCAFT00000001350.3  ENSCAFT00000001363.3  ENSCAFT00000001370.3  ENSCAFT00000001371.3  ENSCAFT00000043181.2  ENSCAFT00000001377.3  ENSCAFT00000001452.3  ENSCAFT00000045486.2  ENSCAFT00000001460.3  ENSCAFT00000001477.3  ENSCAFT00000001482.3  ENSCAFT00000059262.1  ENSCAFT00000056420.1  ENSCAFT00000058351.1  ENSCAFT00000057069.1  ENSCAFT00000001486.2  ENSCAFT00000051674.1  ENSCAFT00000041834.1  ENSCAFT00000053298.1 | ENPP2  TAF2  DSCC1  N/A  DEPTOR  DEPTOR  COL14A1  MRPL13  MRPL13  MTBP  SNTB1  N/A  N/A  N/A  N/A  SYT17  N/A  RNA5SP34  N/A |

**Table S5 continued.**

| CFA | Position (CanFam2.0) | Position (CanFam3.1) | Size of shared ROH (bp) | Number of SNPs in shared parts of ROH regions | Gene ID | Human orthologue |
| --- | --- | --- | --- | --- | --- | --- |
| 18 | 17894276-18452306 | 14904179-15462482 | 558030 | 44 | ENSCAFT00000006466.5  ENSCAFT00000006518.4  ENSCAFT00000006522.3  ENSCAFT00000006618.4  ENSCAFT00000006640.3  ENSCAFT00000042936.1 | EFCAB10  SRPK2  SRPK2  KMT2E  KMT2E  LHFPL3 |
| 19 | 53693452-53694933 | 50671255-50672736 | 1481 | 2 | ENSCAFT00000009048.3 | KIF5C |
| 22 | 3878349-4658978 | 937888-1719379 | 780629 | 54 | ENSCAFT00000044242.2  ENSCAFT00000006902.3  ENSCAFT00000059246.1  ENSCAFT00000055159.1  ENSCAFT00000059202.1  ENSCAFT00000042369.1  ENSCAFT00000053050.1  ENSCAFT00000056915.1  ENSCAFT00000053105.1  ENSCAFT00000006906.1  ENSCAFT00000041883.1  ENSCAFT00000058763.1  ENSCAFT00000050890.1  ENSCAFT00000054936.1 | RNASEH2B  RNASEH2B  N/A  N/A  N/A  RNA5SP243  N/A  N/A  N/A  N/A  N/A  N/A  N/A  N/A |
| 22 | 6532594-6944121 | 3590920-4002242 | 411527 | 28 | ENSCAFT00000007188.3  ENSCAFT00000057604.1  ENSCAFT00000032205.5  ENSCAFT00000050948.1  ENSCAFT00000040200.1  ENSCAFT00000053553.1  ENSCAFT00000048098.1 | N/A  N/A  N/A  N/A  N/A  N/A  N/A |

**Table S5 continued.**

| CFA | Position (CanFam2.0) | Position (CanFam3.1) | Size of shared ROH (bp) | Number of SNPs in shared parts of ROH regions | Gene ID | Human orthologue |
| --- | --- | --- | --- | --- | --- | --- |
| 30 | 26163610-26569134 | 23172022-23578416 | 405524 | 36 | ENSCAFT00000026218.2  ENSCAFT00000026224.2  ENSCAFT00000058781.1  ENSCAFT00000053305.1  ENSCAFT00000059454.1  ENSCAFT00000040202.1  ENSCAFT00000036567.3  ENSCAFT00000026227.2 | PFDN4  AQP9  N/A  N/A  N/A  N/A  LIPC  LIPC |
| 35 | 13073453-13814476 | 10066010-10807066 | 741023 | 60 | ENSCAFT00000048960.2  ENSCAFT00000015340.3  ENSCAFT00000015382.2  ENSCAFT00000048970.1  ENSCAFT00000042932.2  ENSCAFT00000015390.2  ENSCAFT00000015395.3  ENSCAFT00000015470.4  ENSCAFT00000003543.3  ENSCAFT00000052895.1  ENSCAFT00000059119.1  ENSCAFT00000015478.1  ENSCAFT00000015487.2  ENSCAFT00000015494.3  ENSCAFT00000055320.1 | TFAP2A  TFAP2A  GCNT2  C6ORF52  PAK1IP1  PAK1IP1  TMEM14C  MAK  MAK  N/A  N/A  GCM2  SYCP2L  ELOVL2  N/A |
| 36 | 26877913-27332452 | 23851633-24306537 | 454539 | 36 | ENSCAFT00000055195.1  ENSCAFT00000054597.1  ENSCAFT00000034646.1  ENSCAFT00000054860.1  ENSCAFT00000022479.2 | N/A  N/A  N/A  N/A  UBE2E3 |

**Table S5 continued.**

| CFA | Position (CanFam2.0) | Position (CanFam3.1) | Size of shared ROH (bp) | Number of SNPs in shared parts of ROH regions | Gene ID | Human orthologue |
| --- | --- | --- | --- | --- | --- | --- |
| 38 | 23369752-23632983 | 20370158-20633407 | 263231 | 26 | ENSCAFT00000020761.4  ENSCAFT00000044158.1  ENSCAFT00000055893.1  ENSCAFT00000055036.1 | RP11-565P22.6, NOS1AP  RP11-565P22.6, NOS1AP  N/A  N/A |
