# Supplementary material for: Whole genome sequencing identifies missense mutation in MTBP in Shar-Pei affected with Autoinflammatory Disease (SPAID)
Source: BMC Genomics. 2017 May 4;18:348. doi: 10.1186/s12864-017-3737-z (PMC5418765; doi:10.1186/s12864-017-3737-z)
Supplement: Supplementary file 6 — The proportion of gene hits against total number of process hits for genes detected in runs of homozygosity (ROH) regions shared by SPAID (Shar-Pei Autoinflammatory Disease) affected Shar-Pei, which could not be found in control dogs, is shown. PANTHER gene list analysis for biologic processes and pathways was done for human orthologues. (DOCX 16 kb) [file 12864_2017_3737_MOESM6_ESM.docx]

**Table S6. Functional annotation for ROHs exclusively found in SPAID-affected Shar-Pei.** The proportion of gene hits against total number of process hits for genes detected in runs of homozygosity (ROH) regions shared by SPAID (Shar-Pei Autoinflammatory Disease) affected Shar-Pei, which could not be found in control dogs, is shown. PANTHER gene list analysis for biologic processes and pathways was done for human orthologues.

| PANTHER gene ontology terms | Shared ROH regions in SPAID-affected Shar-Pei (%) |
| --- | --- |
| Biological processes |  |
| cellular component organization or biogenesis (GO:0071840) | 6.10 |
| cellular process (GO:0009987) | 26.5 |
| localization (GO:0051179) | 6.10 |
| reproduction (GO:0000003) | 2.00 |
| biological regulation (GO:0065007) | 6.10 |
| response to stimulus (GO:0050896) | 8.20 |
| developmental process (GO:0032502) | 8.20 |
| multicellular organismal process (GO:0032501) | 2.00 |
| locomotion (GO:0040011) | 2.00 |
| metabolic process (GO:0008152) | 26.50 |
| immune system process (GO:0002376) | 6.10 |

**Table S6 continued.**

| PANTHER gene ontology terms | Shared ROH regions in SPAID-affected Shar-Pei (%) |
| --- | --- |
| Pathway |  |
| Triacylglycerol metabolism (P02782) | 16.70 |
| Integrin signalling pathway (P00034) | 16.70 |
| Transcription regulation by bZIP transcription factor (P00055) | 16.70 |
| Inflammation mediated by chemokine and cytokine signaling pathway (P00031) | 16.70 |
| Ubiquitin proteasome pathway (P00060) | 16.70 |
| General transcription regulation (P00023) | 16.70 |
