# Supplementary material for: Whole genome sequencing identifies missense mutation in MTBP in Shar-Pei affected with Autoinflammatory Disease (SPAID)
Source: BMC Genomics. 2017 May 4;18:348. doi: 10.1186/s12864-017-3737-z (PMC5418765; doi:10.1186/s12864-017-3737-z)
Supplement: Supplementary file 7 — Questionnaire for the assessment of health status in investigated Shar-Pei. A description of the project and information consent for dog owners and veterinarians is included. (PDF 160 kb) [file 12864_2017_3737_MOESM7_ESM.pdf]

Prof. Dr. Ottmar Distl and Dr. Julia Metzger
Institute for Animal Breeding and Genetics
University of Veterinary Medicine Hannover
Buenteweg 17p
30559 Hannover, Germany
Phone: +49-511-953-8876; Fax: +49-511-953-8582
Email: ABGLab@tiho-hannover.de

Lab number:
Lab entry:
Confirmation of receipt:

Submission form -
Shar-Pei Autoinflammatory Disease (SPAID) study

Shar-Pei Autoinflammatory Disease (SPAID) is characterized by typical signs of inflammatory processes like recurrent bouts of fever, inflammations of the ear, arthritis and a reddened skin. Due to the circulating inflammatory cells affected dogs can also develop amyloidosis accompanied with severe damage of different organs like kidney, liver, pancreas, heart and intestinal submucosa. This study aims at investigating the genetic cause of SPAID in Shar-Pei. Samples from SPAID-affected and unaffected Shar-Pei dogs are investigated for genetic variants in genes that might be involved in disease development. For accurate assessment of the clinical status it is essential that this form is filled in carefully. All data are kept confidential and thus the ID of the dog or the owner of the dog cannot be inferred.

**Information about the owner**			
Last name:		First name:	
Address:			
Phone / Email:			

**Information about the dog (including pedigree-information)**			
Name & kennel name:			
Sex:	O male O female	date of birth:	
Kennel club:			
Registered no. dog:		Chip-/Tattoo no. dog:	
Registered no. sire:		Chip-/Tattoo no. sire:	
Registered no. dam:		Chip-/Tattoo no. dam:	

**Health status of the dog**	
Skin wrinkling	O little wrinkles O average wrinkles O strong wrinkles
Shar-Pei Fever	O never before O 1-3 times observed O more than 3 times observed

The owner's declaration of agreement

Data and samples of this examination are also used for scientific purposes. All data will be made anonymous and thus the ID of the dog or the owner of the dog cannot be inferred. Herewith, I confirm that the EDTA blood sample of the dog can be used for SPAID research and I agree on the anonymous use of the scientific data.

____
Date and signature of the dog's owner

The veterinarian's declaration of identity verification

I hereby confirm that the sample enclosed has been collected from the dog described above and has been marked immediately by the dog's name and the registered number or the chip number. I agree the additional use of this sample for scientific purposes.

____
Date and signature of the veterinarian

Please see form at the backside!

Please fill in the questionnaire for further research!

**Type and health status**	
**Breed type**	O meat-mouth O bone-mouth
**Wrinkles in the region of the head**	O little O intermediate O much
**Wrinkles in the region of the body**	O little O intermediate O much
**Wrinkles in the region of the legs**	O little O intermediate O much
**Coat type**	O horse coat O brush coat O bear coat
**In the case of pathological examination:** Please add the pathological report	O Yes O No
**Amyloid-positive results** (congo red) in the following organs:	O kidney O liver O pancreas O spleen O others:

**Frequency of occurence**						**Initial age of occurence (years)**							**Comments**
	**1x**	**2x**	**3x**	**>3x**		**-1**	**2**	**3**	**4**	**5**	**6**	**>6**	
**Fever** of unknown origin	O	O	O	O		O	O	O	O	O	O	O	
**Fever** of known origin	O	O	O	O		O	O	O	O	O	O	O	
Please state the potential reason(s) for bouts of fever:													
**Thickened joints** (joint inflammation, arthritis)	O	O	O	O		O	O	O	O	O	O	O	
**Blister-like skin alterations**	O	O	O	O		O	O	O	O	O	O	O	
**Reddening** of the skin	O	O	O	O		O	O	O	O	O	O	O	
**Thickened skin regions** of pasty consistency	O	O	O	O		O	O	O	O	O	O	O	
**Ear inflammations** (recurrent or persistent)	O	O	O	O		O	O	O	O	O	O	O	
**Eye inflammations** (recurrent or persistent)	O	O	O	O		O	O	O	O	O	O	O	
**Diarrhoea** and/or **vomiting** of unknown origin	O	O	O	O		O	O	O	O	O	O	O	
**Tumors**	O	O	O	O		O	O	O	O	O	O	O	
**Type of tumor:**													
**Further diseases:**													
