# Supplementary material for: Whole genome sequencing identifies missense mutation in MTBP in Shar-Pei affected with Autoinflammatory Disease (SPAID)
Source: BMC Genomics. 2017 May 4;18:348. doi: 10.1186/s12864-017-3737-z (PMC5418765; doi:10.1186/s12864-017-3737-z)
Supplement: Supplementary file 8 — Primer sequences and assays for the validation of candidate variants. Kompetitive Allele Specific PCR (KASP) assays were used for six variants whereas further three variants were genotyped by gel electrophoresis on an acrylamide gel. Primer pairs, validation type, amplicon size, annealing temperature and number of PCR cycles are shown. (DOCX 17 kb) [file 12864_2017_3737_MOESM8_ESM.docx]

**Table S7. Primer sequences and assays for the validation of candidate variants.** Kompetitive Allele Specific PCR (KASP) assays were used for six variants whereas further three variants were genotyped by gel electrophoresis on an acrylamide gel. Primer pairs, validation type, amplicon size, annealing temperature and number of PCR cycles are shown.

| CFA | Polymorphism | Forward primer(s) (5’-3’) | Reverse primer (5’-3’) | Validation type | Amplicon size (bp) | Annealing temperature (°C) | Number of cycles |
| --- | --- | --- | --- | --- | --- | --- | --- |
| 1 | *CD79A*:g.112413114insGTGATG | CTGTCTCCTCCTCTCCACAG | CGTAGCTCAGGAAGATGTCG | gel electro-phoresis | 200 | 60 | 36 |
| 6 | *C16orf96*:g.36821482G>T | GGTGAGGATTTTGCTCTGGAGAG-FAM  ATGGTGAGGATTTTGCTCTGGAGAT-VIC | TCTCCTGCCCCCCCAAGGTTT | KASP | 44 | 61 | 26 |
| 6 | *ENSCAFG00000024344*:g.40648375C>T | GGAGAGATCAGCATTGGGACAC-FAM  TGGAGAGATCAGCATTGGGACAT-VIC | GCCCGTATCAAAGASGACCAGGAA | KASP | 57 | 64 | 26 |
| 6 | *RPAP2*:g.56637047delACAA | CACTTCTTTTGCTTGCCTCAG | TTTCCCCTGCTAAATTATTGG | gel electro-phoresis | 259 | 60 | 36 |
| 6 | *TGFBR3*:g.57204844A>G | CACTCACCTGTGTGAGAATAGATGT-FAM  ACTCACCTGTGTGAGAATAGATGC-VIC | AGCACTCCTCACAGGGGCCTT | KASP | 749 | 68 | 29 |
|  |  |  |  |  |  |  |  |

**Table S7** **continued**.

| CFA | Polymorphism | Forward primer(s) (5’-3’) | Reverse primer (5’-3’) | Validation type | amplicon size (bp) | annealing temperature (°C) | Number of cycles |
| --- | --- | --- | --- | --- | --- | --- | --- |
| 13 | *MTBP*:g.19383758G>A | ATCTTAAAACATCAAGGGGTCTATTCG-FAM  GATCTTAAAACATCAAGGGGTCTATTCA-VIC | CAGCATTGTTGTTTGCTGCTTTCTTCATT | KASP | 50 | 61 | 26 |
| 15 | *HCFC2*:g.42583230A>C | TTATTTAAAACACCATCCTTATACCTTCATT-FAM  ATTTAAAACACCATCCTTATACCTTCATG-VIC | GTTGTGTCGTGGATGTGCTAAGGAA | KASP | 57 | 61 | 26 |
| 22 | *CLN5*:g.30574626C>T | GGCTGGCTTGAACAGTCCACG-FAM  GGCTGGCTTGAACAGTCCACA-VIC | GTAAAGCGGGACAATGAAACAGGAATTTA | KASP | 58 | 61 | 32 |
| 37 | *OSGEPL1*:g.502225delCTTGTGCAA | GTTCCGGACAGTTTGTTTCTTC | AACAGTTTTCCAGGATCAAAGC | gel electro-phoresis | 156 | 60 | 36 |
