# Supplementary material for: Whole genome sequencing identifies missense mutation in MTBP in Shar-Pei affected with Autoinflammatory Disease (SPAID)
Source: BMC Genomics. 2017 May 4;18:348. doi: 10.1186/s12864-017-3737-z (PMC5418765; doi:10.1186/s12864-017-3737-z)
Supplement: Supplementary file 9 — Primers for sequencing MTBP complementary DNA. The gene regions, product sizes and annealing temperatures are shown (DOCX 14 kb) [file 12864_2017_3737_MOESM9_ESM.docx]

**Table S8. Primers for sequencing *MTBP* complementary DNA.** The gene regions, product sizes and annealing temperatures are shown.

| Gene | Gene region | Forward primer (5’-3’) | Reverse primer (5’-3’) | Product size (bp) | Annealing temperature (°C) |
| --- | --- | --- | --- | --- | --- |
| *MTBP* | exon 1-8 | GTACCTGCTCCTCGTGATCTG | CTTGTTGGTTAGAATGCAAGG | 820 | 60 |
| *MTBP* | exon 3-11 | CCTGGTTCCAAGAAGTGGTTC | ACACGGCAATACAAAAAGTGC | 855 | 60 |
| *MTBP* | exon 10-17 | TTGTTGTTGGAGCAGATTTCC | ACCCTGAAGTTTCCGAGGAC | 924 | 60 |
| *MTBP* | exon 18-22 | TCCGTTATGAAACCCAAACC | CAACCAAGGAAGGAGGTAGC | 738 | 60 |
| *MTBP* | exon 12-18 | AAGGACAGAAGAAGCCAAACTG | CATAACGGATGAGACGAGACTG | 740 | 60 |
| *MTBP* | exon 20-22 | GCTCATGGATGTTTCACTGC | TGAGGCAATGTTGGAATTTG | 818 | 60 |
